# Characteristics and Risk Factors for Mortality in Critically Ill Patients with COVID-19 Receiving Invasive Mechanical Ventilation: The Experience of a Private Network in Sao Paulo, Brazil

**DOI:** 10.2478/jccm-2022-0015

**Published:** 2022-08-12

**Authors:** Eduardo Atsushi Osawa, Alexandre Toledo Maciel

**Affiliations:** 1 Imed Group, Research Department, Sao Paulo Brazil; 2 Adult Intensive Care Unit, São Camilo Hospital, Sao Paulo Brazil

**Keywords:** coronavirus, COVID-19, mortality, mechanical ventilation, body mass index, barotrauma

## Abstract

**Introduction:**

The use of invasive mechanical ventilation (IMV) in COVID-19 represents in an incremental burden to healthcare systems.

**Aim of the study:**

We aimed to characterize patients hospitalized for COVID-19 who received IMV and identify risk factors for mortality in this population.

**Material and Methods:**

A retrospective cohort study including consecutive adult patients admitted to a private network in Brazil who received IMV from March to October, 2020. A bidirectional stepwise logistic regression analysis was used to determine the risk factors for mortality.

**Results:**

We included 215 patients, of which 96 died and 119 were discharged from ICU. The mean age was 62.7 ± 15.4 years and the most important comorbidities were hypertension (62.8%), obesity (50.7%) and diabetes (40%). Non-survivors had lower body mass index (BMI) (28.3 [25.5; 31.6] vs. 31.2 [28.3; 35], p<0.001, and a shorter duration from symptom onset to intubation (8.5 [6.0; 12] days vs. 10 [8.0; 12.5] days, p = 0.005). Multivariable regression analysis showed that the risk factors for mortality were age (OR: 1.07, 95% CI: 1.03 to 1.1, p < 0.001), creatinine level at the intubation date (OR: 3.28, 95% CI: 1.47 to 7.33, p = 0.004), BMI (OR: 0.91, 95% CI: 0.84 to 0.99, p = 0.033), lowest PF ratio within 48 hours post-intubation (OR: 0.988, 95% CI: 0.979 to 0.997, p = 0.011), barotrauma (OR: 5.18, 95% CI: 1.14 to 23.65, p = 0.034) and duration from symptom onset to intubation (OR: 0.76, 95% CI: 0.76 to 0.95, p = 0.006).

**Conclusion:**

In our retrospective cohort we identified the main risk factors for mortality in COVID-19 patients receiving IMV: age, creatinine at the day of intubation, BMI, lowest PF ratio 48-hours post-intubation, barotrauma and duration from symptom onset to intubation.

## Introduction

The coronavirus disease [COVID-19] was first reported in Wuhan, China and spread rapidly across the globe causing millions of deaths worldwide. Patients hospitalized for COVID-19 are at high risk of developing acute respiratory failure and receiving invasive mechanical ventilation [IMV]. In an effort to understand a novel disease and gain knowledge from other centres, multiple epidemiological cohorts have been carried out in different settings. Nevertheless, a striking variability in the mortality rates were reported among those requiring mechanical ventilation [1, 2].

The understanding of which factors are associated with greater mortality in COVID-19 patients receiving mechanical ventilation would enable clinicians to perform better treatment decisions to mitigate the progression of a severe illness. Previously, studies assessing COVID-19 mortality did not incorporate variables specific to intensive care or were restricted to a non-generalizable population [3, 4]. Moreover, the majority of studies aiming to evaluate mortality in COVID-19 patients addressed this issue in a larger population of hospitalized patients, but few of them did so in the subgroup of patients receiving IMV.

Thus, we aimed to describe patients admitted for COVID-19 respiratory infection who received IMV in a private network in Sao Paulo, Brazil and determine which factors were associated with increased mortality.

## Materials and Methods

### Study design and participants

**Fig.1:**
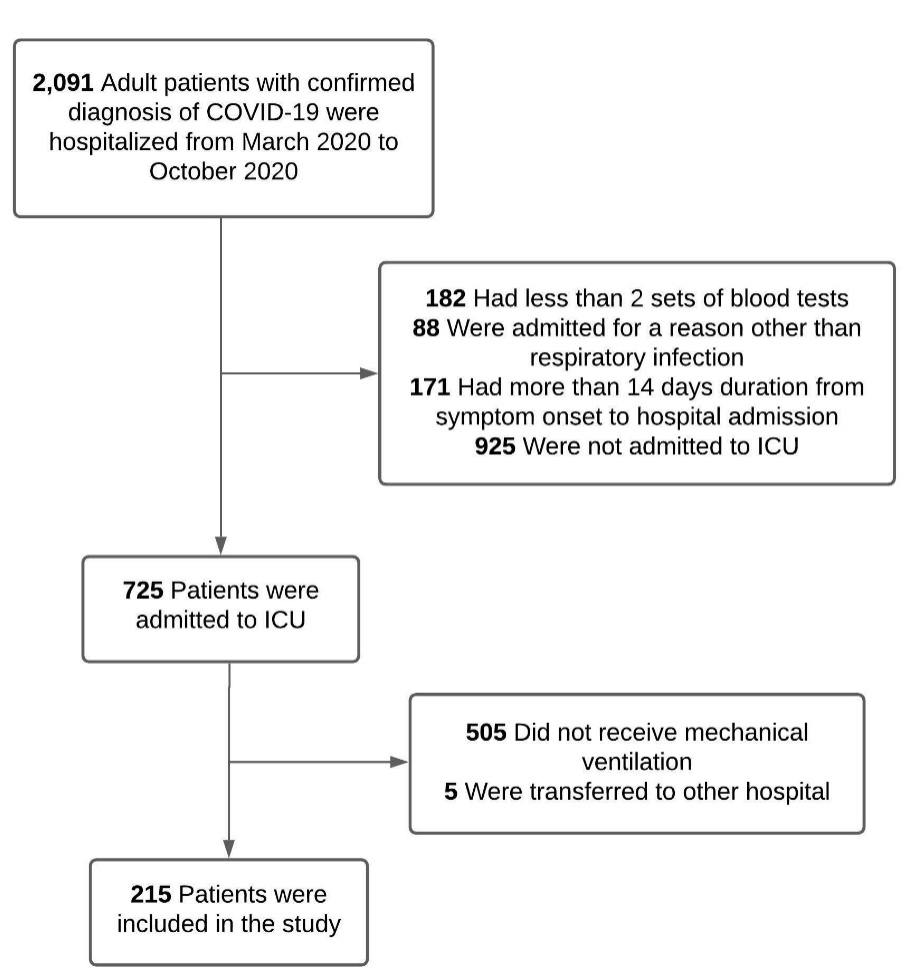
Flowchart of the study enrolment. ICU: Intensive Care Unit.

We performed a retrospective cohort study in a private network in Sao Paulo, Brazil (Hospital Sao Camilo) comprising 3 tertiary care hospitals (Pompeia, Santana and Ipiranga Units). We identified consecutive adult patients (aged ≥ 18 years) admitted to the Intensive Care Unit (ICU) of one of the 3 hospitals from March 18, 2020 to October 26, 2020 who required IMV during the course of their stay. The decision to intubate was made at the discretion of the treating team and was not supported by any prediction score. Enrolled patients had the diagnosis of SARS-CoV-2 infection confirmed by RT-PCR of nasopharyngeal or oropharyngeal swab sample. We excluded patients who did not have at least 2 sets of blood tests obtained during hospital stay, those whose symptom onset commenced more than 14 days prior to hospital admission, patients who were transferred to other hospital system and patients admitted for reasons other than respiratory infection (Figure 1).

In our institution, a protective ventilation strategy was adopted through the use of either pressure-controlled or volume-controlled ventilation aiming to achieve the following parameters: a tidal volume of 6ml/ kg, FiO2 adjustment to achieve an oxygen saturation between 88 and 92%, a plateau pressure lower than 30 mmHg, and a driving pressure lower than 15 cm H2O. Decremental PEEP titration was performed guided by driving pressure. We used the predicted body weight (PBW) to normalize the tidal volume and reduce the mechanical lung strain, particularly in obese patients. In patients requiring neuromuscular blockade, a non-depolarizing agent was used: rocuronium (8 to 12 mcg/ kg/minute) or cisatracurium (1 to 3 mcg/kg/minute) and the recommended maximum duration of infusion was 96 hours. Prone positioning was performed on the first 7 days post-intubation whenever the pO_2_/FiO_2_ ratio was lower than 150, for the duration of 16 hours. All patients received pharmacological thromboprophylaxis unless a formal contra-indication was present.

With regards to pharmacological treatment, our institutional protocol underwent temporal modifications to incorporate evidence emerging from clinical trials. After the publication of the RECOVERY study [5], corticosteroid was prescribed for all hospitalized patients requiring oxygen therapy. Hydroxychloroquine was administered as a compassionate therapy early in the pandemic for hospitalized patients and stopped being used after the publication of the Coalition Covid-19 Brazil I study [6]. Tocilizumab was administered for hypoxemic patients fulfilling the criteria for cytokine storm (elevated levels of C-reactive protein, ferritin, lactate dehydrogenase and interleukin 6) in the absence of bacterial infection.

The primary outcome of our study was ICU mortality. Secondary outcomes included duration of IMV and ventilator-free days, worst PaO2/FiO2 ratio and PEEP level within 48 hours post intubation, use of pharmacological and intensive care support therapies, occurrence of barotrauma, tracheostomy and reintubation rates, and ICU and hospital length of stay.

Barotrauma was defined as the diagnosis of pneumothorax, pneumomediastinum or subcutaneous emphysema occurring after hospitalization.

### Ethics

The study was approved by the local Ethics Committee of São Camilo Hospital and the study procedures were conducted in accordance with its ethical standards and with the Helsinki Declaration. Written informed consent was waived due to the retrospective nature of the study.

### Data collection

For all patients, we collected data on baseline characteristics including demographics, comorbidities, peripheral oxygen saturation at hospital admission, height, weight, date of symptom onset, and outcome (discharge, death or transfer). Obesity was defined as a body mass index (BMI) greater than 30 kg/m2. We also recorded SAPS 3 score, date of intubation, and dates of admission and discharge. During ICU stay, we obtained data on the use of non-invasive ventilation strategies, medical therapies, and requirement of organ support therapies such as vasopressor agents, prone positioning, neuromuscular blockade, renal replacement therapy (RRT) and veno-venous ECMO. The modality of RRT used was continuous veno-venous hemofiltration with a polycarbonate membrane filter and citokine adsorber was not used.

Laboratory data of the first 14 days following hospitalization were extracted from electronic medical records. Daily measurements of the following biomarkers were obtained: C-reactive protein (CRP), lactate dehydrogenase (LDH), D-dimer and creatinine levels; lymphocyte, neutrophil and platelet counts. We calculated two parameters: the neutrophil-to-lymphocyte ratio (NLR) was obtained by dividing the absolute neutrophil count by the absolute lymphocyte count, and lymphocyte-C-reactive-protein ratio (LCR) was calculated by dividing lymphocyte count by the CRP level. Blood samples were routinely obtained once daily. In cases of more than one set of blood tests obtained on a single day, the worst value was considered. In patients who required RRT, we recorded serum creatinine levels up to the last measurement before the therapy commenced. We did not record creatinine levels of patients who had chronic kidney disease on-dialysis. Also, in patients who received tocilizumab, we obtained serum CRP levels until the day this therapy had been administered.

### Statistical analysis

Continuous variables were presented as means and standard deviations or medians and interquartile ranges (median [quartile 1; quartile 3]), as appropriate. Normal distribution of continuous variables was assessed with the Kolmogorov-Smirnov test. Categorical variables were summarized as counts and percentages. No imputation was made for missing data.

Chi-squared test or Fisher exact test was used to compare categorical variables as appropriate. Kruskal-Wallis or Mann-Whitney rank sum test was used to compare non-parametric continuous variables and t-student test was used to compare parametric continuous variables.

A bidirectional stepwise logistic regression analysis was undertaken to determine the risk factors for mortality. Variables that were significantly associated with the outcome in the univariate analysis were included in the multivariate model according to the following criteria: clinical relevance, lack of collinearity and missing data < 15% of cases. We used 80% of our study population to develop the model and applied it to the remaining 20% to validate its performance. Overall goodness of fit was verified by Akaike Information Criterion and discrimination of the model was evaluated by receiver-operator characteristics (ROC) curve of predicted probability.

All statistical tests were 2-tailed, and a P-value < 0.05 was considered statistically significant. Statistical tests were performed using R version 4.0.2.

## Results

**Fig. 2:**
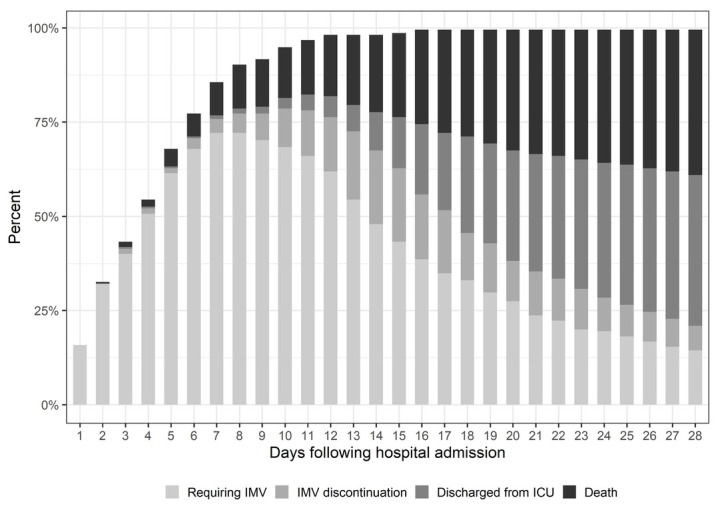
Daily distribution of patients according to study events. ICU: Intensive Care Unit; IMV: invasive mechanical ventilation.

We included 215 patients in our study, of which 96 died and 119 were discharged from ICU. The baseline characteristics of study patients are demonstrated in table 1. Patients were predominantly male (67.9%) and the mean age was 62.7 ± 15.4 years. The most important comorbidities were hypertension (62.8%), obesity (50.7%) and diabetes (40%). The body mass index (BMI) among participants who deceased was 28.3 [25.5; 31.6] kg/m^2^ and 31.2 [28.3; 35] kg/m^2^ in those who survived, p < 0.001. The median duration from symptom onset to hospital admission was 7 [5; 9] days and the median SAPS 3 score was 49 [44; 55]. The distribution and timing of outcomes (initiation of IMV, IMV discontinuation, ICU discharge and death) are represented in figure 2.

**Table 1:** Baseline characteristics of study patients.

Variable	Overall (N = 215)	Discharged from ICU (N = 119)	Died in ICU (N = 96)	P-value
Female gender	69 (32.1)	34 (28.6)	35 (36.5)	0.242
Age, years	62.7 ± 15.4	56.9 ± 13.9	69.9 ± 14.2	< 0.001
Comorbidities				
Hypertension	135 (62.8)	68 (57.1)	67 (69.8)	0.066
Diabetes	86 (40.0)	41 (34.5)	45 (46.9)	0.070
COPD	15 (7.0)	3 (2.5)	12 (12.5)	0.006
Smoking	3 (1.4)	0 (0.0)	3 (3.1)	0.087
Obesity	109 (50.7)	75 (63.0)	34 (35.4)	<0.001
Heart failure	15 (7.0)	2 (1.7)	13 (13.5)	< 0.001
Cirrhosis	1 (0.5)	0 (0.0)	1 (1.0)	0.447
Stroke	6 (2.8)	0 (0.0)	6 (6.2)	0.007
Bariatric surgery	2 (0.9)	2 (1.7)	0 (0.0)	0.503
Hyperlipidemia	47 (21.9)	23 (19.3)	24 (25.0)	0.325
Dementia	5 (2.3)	1 (0.8)	4 (4.2)	0.175
Autoimmune disorder	1 (0.5)	1 (0.8)	0 (0.0)	1.000
HIV	4 (1.9)	3 (2.5)	1 (1.0)	0.630
Solid neoplasm	5 (2.3)	3 (2.5)	2 (2.1)	1.000
Hematological neoplasm	3 (1.4)	1 (0.8)	2 (2.1)	0.587
Asthma	12 (5.6)	8 (6.7)	4 (4.2)	0.554
Non-dialysis CKD	14 (6.5)	6 (5.0)	8 (8.3)	0.408
CKD on dialysis	6 (2.8)	2 (1.7)	4 (4.2)	0.411
Coronary artery disease	24 (11.2)	10 (8.4)	14 (14.6)	0.192
Duration from symptom onset to hospital admission, days	7 [5; 9]	7 [5; 9]	6 [5; 8]	0.084
BMI	30.1 [26.6; 34.2]	31.2 [28.3; 35]	28.3 [25.5; 31.6]	<0.001
SpO2 at hospital presentation	91 [86; 95]	92 [88; 96]	90 [82.5; 95]	0.075
SAPS 3 score	49 [44; 55]	47 [42; 51]	54 [48; 59]	< 0.001
Duration from hospital admission to intubation date	3 [1; 5]	4 [1; 6]	3 [1; 5]	0.107

BMI: Body mass index; CKD: Chronic kidney disease; COPD: Chronic obstructive pulmonary disease; HIV: Human immunodeficiency virus; ICU: Intensive Care Unit; SAPS: Simplified Acute Physiology Score; SpO2: Arterial oxygen saturation.

**Fig. 3:**
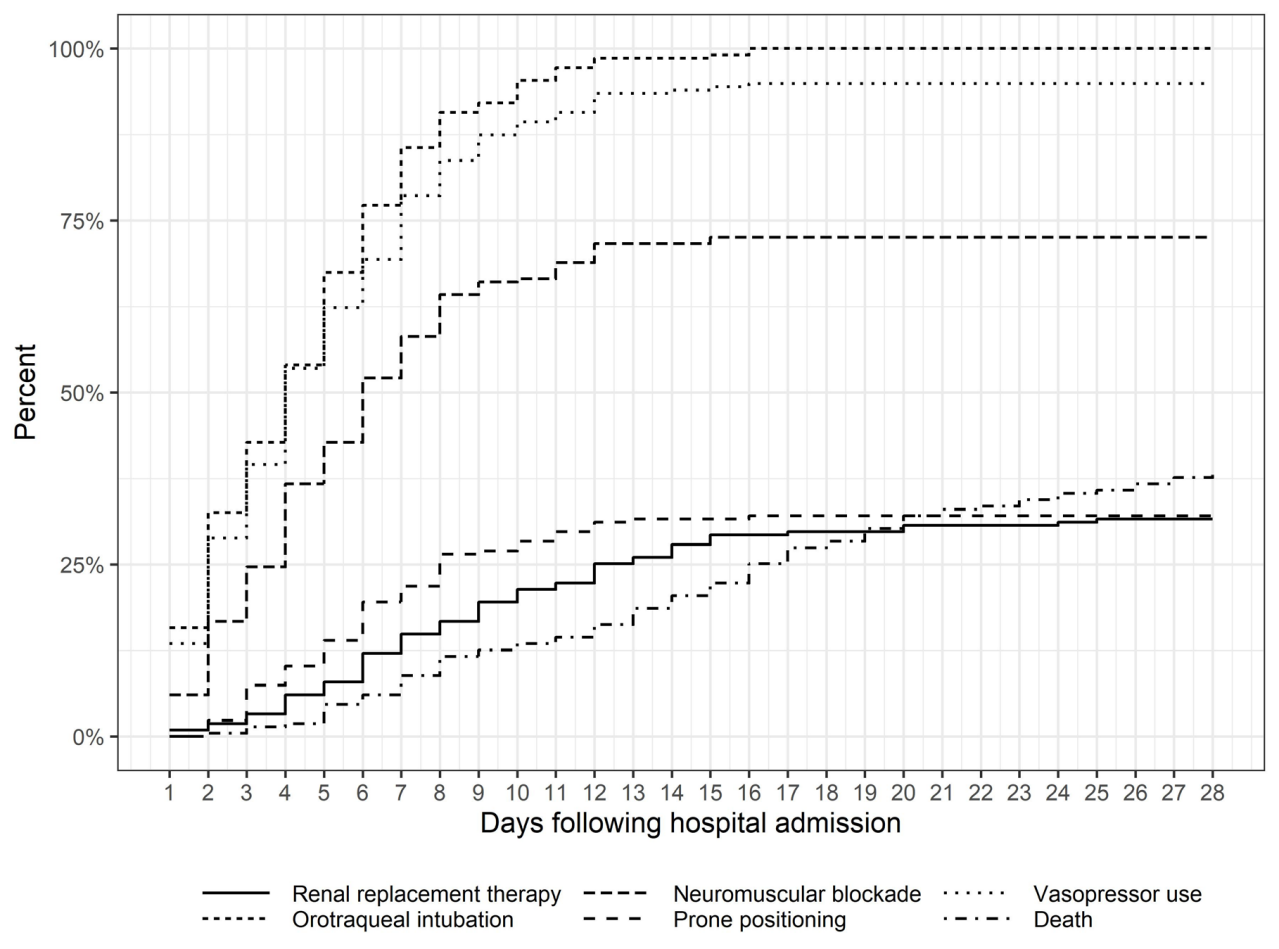
Cummulative percentage of study outcomes within the first 28 days of hospitalization.

Also, the cumulative proportion of patients requiring intensive care support therapies over the first 28 days of hospitalization was plotted against death in figure 3.

We analysed the distribution of measurements of laboratory biomarkers at 2 timepoints: hospital admission (table 2) and date of intubation (table 3). We observed that NLR and serum creatinine differed between groups at both timepoints. Furthermore, at hospital admission, those who died had greater D-dimer concentration and lower lymphocyte count in comparison to those who were discharged from ICU.

**Table 2:** Laboratory biomarkers measurements obtained at hospital admission.

Variable	Overall (N = 215)	Discharged from ICU (N = 119)	Died in ICU (N = 96)	P-value
CRP	113 [55; 201]	112 [44; 184]	118 [58; 221]	0.165
LDH	380 [281; 510]	364 [269; 460]	416 [294; 536]	0.177
Creatinine	1.0 [0.9; 1.4]	1.0 [0.8; 1.2]	1.1 [0.9; 1.6]	0.002
Lymphocyte count	904 [651; 1266]	932 [718; 1284]	829 [545; 1244]	0.021
Neutrophil count	5050 [3462; 7480]	4812 [3407; 6860]	5346 [3497; 9044]	0.301
Platelet count	180 [138; 232]	186 [142; 231]	172 [136; 240]	0.356
D-dimer	0.6 [0.4; 1.0]	0.5 [0.3; 0.8]	0.7 [0.4; 1.7]	0.003
LCR	8 [4; 18]	9 [4; 25]	6 [3; 14]	0.019
NLR	6 [3; 11]	5 [3; 9]	7 [4; 14]	0.005

CRP: C-reactive protein; LDH: lactate dehydrogenase; LCR: lymphocyte-to-CRP ratio; NLR: neutrophil-to-lymphocyte ratio.

**Table 3:** Laboratory biomarkers obtained at the date of intubation.

Variable	Overall (N = 215)	Discharged from ICU (N = 119)	Died in ICU (N = 96)	P-value
CRP	221 [143; 293]	199 [139; 270]	235 [155; 329]	0.069
LDH	493 [400; 643]	485 [387; 573]	528 [429; 676]	0.073
Creatinine	1.1 [0.8; 1.5]	1.0 [0.8; 1.3]	1.3 [1.0; 2.0]	< 0.001
Limphocyte count	866 [560; 1238]	884 [586; 1264]	793 [520; 1142]	0.310
Neutrophil count	8039 [5322; 11509]	6850 [5054; 10847]	10014 [6690; 12617]	0.005
Platelet count	228 [179; 300]	240 [196; 302]	206 [161; 284]	0.025
D-dimer	1.1 [0.6; 2.3]	0.9 [0.6; 1.6]	1.2 [0.7; 2.4]	0.301
LCR	4 [3; 8]	4 [3; 8]	4 [2; 7]	0.124
NLR	10 [6; 16]	8 [6; 13]	12 [7; 19]	0.004

CRP: C-reactive protein; LDH: lactate dehydrogenase; LCR: lymphocyte-to-CRP ratio; NLR: neutrophil-to-lymphocyte ratio.

In our population, 53.5% of patients used non-invasive ventilation (NIV) and 34.9% used high-flow nasal cannula (HFNC) prior to IMV initiation. The proportion of patients using the aforementioned non-invasive devices before intubation was similar between groups (Table 4) After intubation, 69 patients (32.1%) patients underwent prone positioning and 158 (73.5%) received neuromuscular blockade. Within the period of 48 hours post-intubation, the P/F ratio was lower in those who died compared to those who survived, but the highest PEEP level was similar between groups. We also found that patients who died had a greater requirement of renal replacement therapy (56.2% vs 14.3%, P < 0.001) and a higher incidence of barotrauma (14.6% vs 8.4%, P = 0.007). Seven patients (3.3%) used veno-venous ECMO, two of them in the group who survived and 5 patients in the group who died in ICU (p = 0.010). ECMO was initiated after a median of 7 [6.0; 8.5] days from the day of intubation.

**Table 4:** Suportive treatment received while in ICU and clinical outcomes.

Variable	Overall (N = 215)	Discharged from ICU (N = 119)	Died in iCU (N = 96)	P-value
Non-invasive strategies prior to IMV				
NIV	115 (53.5)	69 (58.0)	46 (47.9)	0.182
HFNC	75 (34.9)	38 (31.9)	37 (38.5)	0.386
Prone positioning	69 (32.1)	36 (30.3)	33 (34.4)	0.619
Neuromuscular blockade	158 (73.5)	88 (73.9)	70 (72.9)	0.988
Nitric oxide	6 (2.8)	2 (1.7)	4 (4.2)	0.016
ECMO	7 (3.3)	2 (1.7)	5 (5.2)	0.010

Worst parameters within 48 hours post-IMV				
PEEP	12 [10; 14]	12 [10; 14]	12 [10; 14]	0.852
P/F ratio	126 [93.5; 162]	140 [104; 174]	110 [87; 138]	<0.001
Renal replacement therapy	71 (33)	17 (14.3)	54 (56.2)	<0.001
Vasopressor use	206 (95.8)	113 (95.0)	93 (96.9)	0.722

Pharmacological treatment				
Tocilizumab	31 (14.4)	25 (21)	6 (6.3)	0.004
Corticosteroid	109 (50.7)	64 (53.8)	45 (46.9)	0.384
Hydroxycloroquine	71 (33)	46 (38.7)	25 (26)	0.070
Tracheostomy	39 (18.1)	20 (16.8)	19 (19.8)	0.699
Reintubation	22 (10.2)	10 (8.4)	12 (12.5)	0.448
Barotrauma	18 (8.4)	4 (3.4)	14 (14.6)	0.007
Duration of NIV before IMV, days	1 [1.0; 3.0]	2 [1.0; 3.0]	2 [1.0; 3.0]	0.861
Duration of HFNC before IMV, days	1 [1.0; 2.0]	1 [1.0; 3.0]	1 [1.0; 2.0]	0.677
Duration of IMV, days	10 [7; 18]	10 [7; 16]	11 [6; 19]	0.876
ICU length of stay, days	15 [10; 24]	17 [11.5; 25.5]	13 [7; 21.2]	0.002
Hospital length of stay, days	21 [15; 30]	27 [19.5; 39.5]	14.5 [7; 22.2]	<0.001
Days from hospital admission to intubation	3 [1.0; 5.0]	4 [1.0; 5.5]	3 [1.0; 5.0]	0.111
Days from symptom onset to intubation	9 [7 – 12]	10 [8.0 – 12.5]	8.5 [6 – 12]	0.005
Ventilator-free days	9 [3; 16]	15 [11; 23.5]	2 [0; 4.0]	<0.001

ECMO: Extracorporeal membrane oxygenation; HFNC: High-flow nasal cannula; ICU: Intensive Care Unit; IMV: mechanical ventilation; NIV: Non-invasive ventilation; PEEP: Positive end-expiratory pressure; P/F ratio: PaO2/FiO2 ratio.

With regards to pharmacological treatment, 31 patients (14.4%) received tocilizumab and the proportion of use was higher in those who were discharged from ICU (21% vs 6.3%, p = 0.004). Around half of our population used corticosteroid and a third used hydroxychoroquine (Table 4)

The median duration of IMV was 10 [7; 18] days. Participants who were discharged from ICU had longer ventilator free-days as compared to those who deceased (23 [16; 35] days vs. 11 [5; 19] days, p < 0.001). We observed that patients who died had a shorter duration from symptom onset to intubation (8.5 [6; 12] days vs. 10 [8.0; 12.5] days, p = 0.005). Twenty-two patients (10.2%) underwent reintubation, but their number of ventilator-free days was similar as compared to the duration of non-reintubated patients (7.5 [2.25; 15.8] ventilator free-days versus 9.0 [3.0; 16] ventilator-free days, respectively, p = 0.848). Tracheostomy was performed in 39 patients (18.1%) after a median of 20 [18; 22] days from ICU admission. Patients who had a tracheostomy inserted had 14 [2.5; 28.5] ventilator-free days versus 9 [3; 14] days in patients who did not undergo tracheostomy, p = 0.178. In addition, patients who died had shorter ICU and hospital length of stay as compared to those who survived (Table 4)

Patients with a BMI greater than 30 kg/m^2^ had similar occurrence of barotrauma as compared to non-obese patients (7 patients [6.4%] versus 11 patients [10.6%], respectively, p = 0.399) and similar duration of mechanical ventilation (11 [6; 19;5] days versus 10 [7; 16] days, respectively, p = 0.532).

**Fig. 4:**
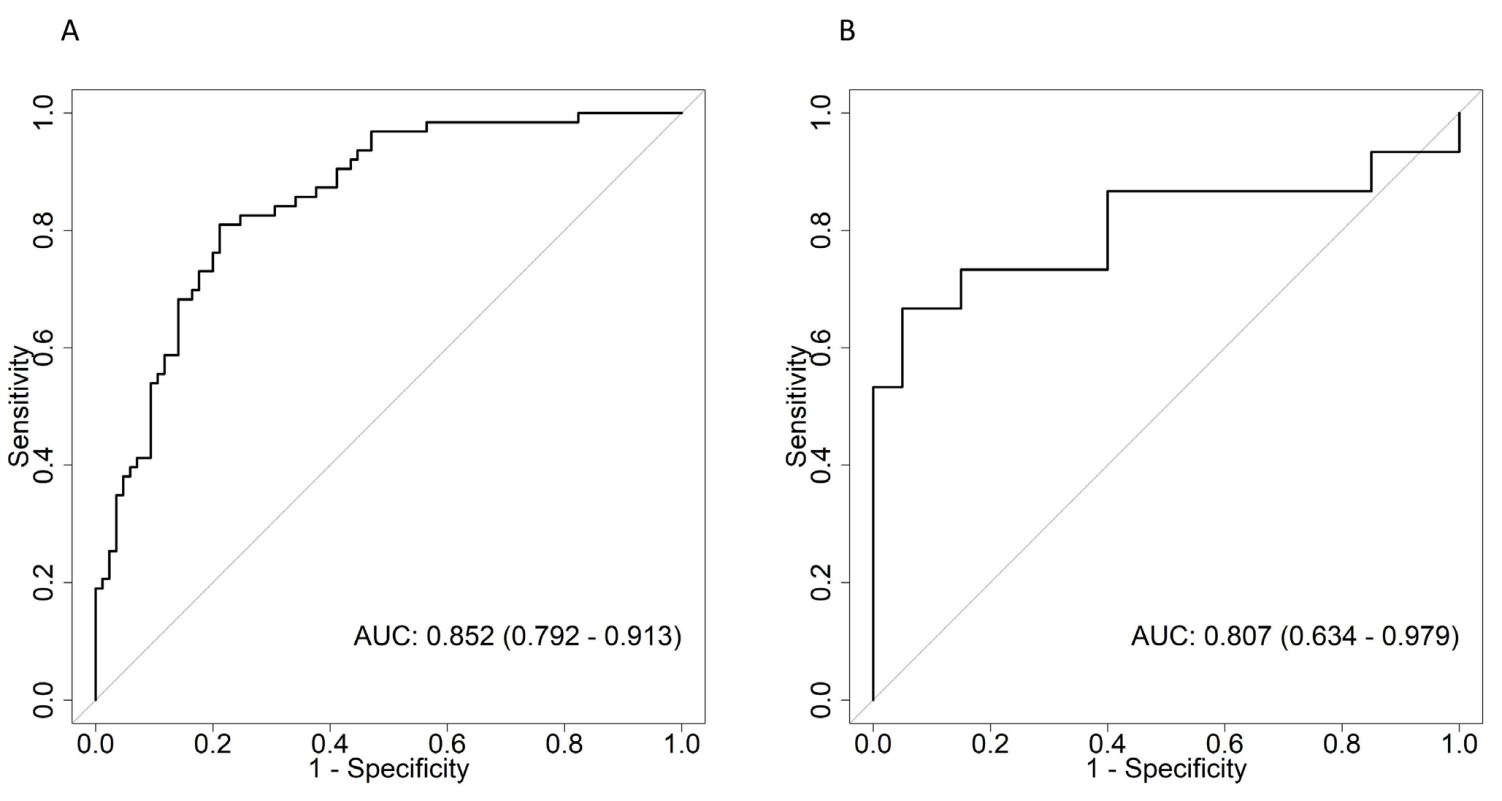
ROC curves on the multivariable logistic regression model to identify risk factors for mortality (A) Development cohort (B) Validation cohort. AUC: area under curve; ROC: Receiver Operating Characteristic.

Our multivariable regression model demonstrated that older age (OR: 1.07, 95% CI: 1.03 to 1.1, p < 0.001), higher creatinine at the intubation date (OR: 3.28, 95% CI: 1.47 to 7.33, p = 0.004), lower BMI (OR: 0.91, 95% CI: 0.84 to 0.99, p = 0.033), lowest PF ratio within 48 hours post-intubation (OR: 0.988, 95% CI: 0.979 to 0.997, p = 0.011), occurrence of barotrauma (OR: 5.18, 95% CI: 1.14 to 23.65, p = 0.034) and lower duration from symptom onset to intubation date (OR: 0.76, 95% CI: 0.76 to 0.95, p = 0.006) were risk factors for mortality.(Table 5) The area under curve (AUC) for the development cohort was 0.852 (95% CI: 0.792 to 0.913) and for the validation cohort was 0.807 (95% CI: 0.634 to 0.979) (Figure 4)

**Table 5:** Risk factors for in-hospital mortality.

Variable	Estimate	Standard Error	Odds ratio (OR)	95% CI	P value
Age, per year	0.065	0.016	1.07	1.47 to 7.33	< 0.001
Creatinine at intubation day	1.188	0.410	3.28	0.84 to 0.99	0.004
BMI	-0.089	0.042	0.91	0.979 to 0.997	0.033
Lowest PF ratio 48 hours post intubation	-0.012	0.005	0.988	1.14 to 23.65	0.011
Barotrauma	1.646	0.774	5.18	0.76 to 0.95	0.034
Duration from symptom onset to intubation	-0.161	0.059	0.85	1.03 to 1.1	0.006

BMI: Body mass index; PF ratio: PaO2/FiO2 ratio.

## Discussion

We reported the clinical characteristics, resource use, and the clinical outcomes of critically ill patients admitted for COVID-19 who received IMV in a private network in São Paulo, Brazil. Also, we documented the values of laboratory biomarkers obtained at two time-points: hospital admission and intubation date. Finally, we built a multivariable regression model aiming to identify the risk of mortality during ICU stay.

The majority of our population was composed by male patients with hypertension and the overall ICU mortality rate was 45%, similar to previous studies [7, 8, 9, 10, 11].

In our study, the BMI measurement was lower in participants who died and a reduced BMI was associated with higher mortality in the multivariable regression model. A similar pattern was demonstrated by an observational study conducted in the USA [12]. This inverse relationship, termed obesity paradox, has also been suggested by a study showing that ICU patients with moderate obesity had a lower risk of death [13]. A multicentre cohort performed in mechanically ventilated patients in the Netherlands reported lower 90-day mortality rates in the obese group as compared to normal and overweight patients, although this difference was no longer evident in the adjusted analysis [14].

Among patients who died in our ICU, even though the duration from hospital admission to intubation was similar to those who survived, they had shorter time length from symptom onset to intubation date. This finding is in line with a study performed in Italian ICUs [7]. Interestingly, a greater 28-day mortality linked with shorter time from viral symptom onset to ICU admission and a greater occurrence of acute kidney and myocardial injury has been previously reported [15]. These observations raise the hypothesis that severe outcomes in patients with COVID-19 are related to earlier disease progression and rapid multiorgan derangement.

Invasive mechanical ventilation is the main driver of incremental healthcare burden in patients hospitalized for COVID-19 [16]. Early in the pandemic in particular, the interpretation of data on resource use was affected by a significant proportion of patients who were still hospitalized at the moment when studies were published [8, 9, 17, 18, 19]. Recent studies have documented variable rates of mortality and use of organ support therapies, and this was the case even at the same jurisdiction [20, 21, 22]. Several factors were implicated as the cause for this variation such as the strain faced by health systems and the unequal pattern of clinical decision making and strategies of ventilation [23]. Neuromuscular blocking agents were used in a large proportion of our patients, as also reported by some studies [24, 25], but in contrast with others [9, 22]. We hypothesize that such practice was derived from human resource shortage, an issue also encountered by other investigators [25]. Also, ECMO was a scarce resource in our centre during the pandemic. Thus, the higher use of ECMO in patients who died may be attributed to greater disease severity compounded by delayed initiation. Furthermore, the resource shortage caused by the pandemic may have resulted in the use of inhaled nitric oxide in few of our patients. This therapy is associated with immediate oxygenation improvement which can delay respiratory deterioration until advanced resources become available [26]. Similar to ECMO, the statistically significant difference with a higher use in patients who died in ICU is probably also a reflection of disease severity.

The observation period of our study commenced from the stage when a novel disease was being understood and was followed by the emergence of new evidence and rapid practice change. Despite the potential influence of such modifications on the population’s trajectory, our model addressed parameters obtained mostly at hospital admission and around the intubation date. Given that these timepoints may not be directly affected by the new therapies, our findings remain informative for clinicians treating patients with COVID-19 who receive IMV. Of note, tocilizumab was prescribed in a greater proportion in those patients who survived. The benefit of tocilizumab in COVID-19 is a topic of debate in the literature due to heterogeneity of study outcomes and the existence of multiple factors affecting patient’s response such as disease severity and SARSCoV-2 variants [27]. A systematic review and meta-analysis of observational studies showed decreased mortality in COVID-19 patients treated with tocilizumab [28]. Also, a recent randomized controlled trial demonstrated improved outcomes and survival in critically ill patients who received interleukin-6 receptor antagonists [29].

Renal replacement therapy was performed in a third of our population, a finding identical to previous investigations [10, 30]. Moreover, a greater serum creatinine value measured at the date of intubation was associated with greater mortality in our model. The close relationship between acute kidney injury and mechanical ventilation has been examined by a retrospective cohort describing that the time of intubation and initiation of RRT were highly clustered [31]. Another cohort demonstrated that invasive mechanical ventilation was more frequent in those who developed AKI, and such effect was higher by AKI stage [32].

In our study, barotrauma was more frequent in the group who died and was included in our multivariable model as a risk factor for mortality. This association has also been documented by other retrospective studies reporting a greater occurrence of barotrauma in mechanically ventilated patients and a higher mortality among those who developed this complication [33, 34, 35, 36]. During mechanical ventilation, positive pressure ventilation and elevated pressures increase the risk of alveolar rupture [37]. In our population, however, barotrauma occurred despite the implementation of a lung protective ventilation strategy. A case-control study showed that ventilated COVID-19 patients who developed barotrauma had low median values of peak inspiratory pressure, plateau pressure and tidal volume 24 hours prior to this complication [38]. In this context, a possible explanation for barotrauma occurrence was the presence of greater diffuse alveolar damage and virus-related factors such as microthrombosis and angiogenesis [39, 40]. Moreover, it has been demonstrated that the incidence of barotrauma is higher in patients with COVID-19 in comparison to other causes of acute respiratory distress syndrome [41]. Barotrauma occurrence may, therefore, be interpreted as an epiphenomenon of a greater lung destruction induced by COVID-19 with its consequent greater susceptibility to additional damage related to positive pressure ventilation [42] despite the use of a protective approach [38].

Our study carries some limitations. First, it was an observational study performed in a single private health system. Nevertheless, our hospitals are located in one of the most hardly affected regions of the globe and the observation period included different stages of the pandemic. Second, for logistic reasons, we were unable to record mechanical ventilation parameters such as tidal volume, plateau pressure, peak inspiratory pressure, driving pressure, compliance, or mechanical power. Also, multiple changes in ventilator settings were performed over a prolonged ICU stay to accommodate different disease stages, a complex scenario which thwarted the acquisition of a single estimate that would be representative of the entire stay. Third, our population was composed by patients placed on IMV in the midst of a pandemic. While the decision to ventilate is not uniform even under non-overwhelming circumstances [43, 44], this matter may have been aggravated by the augmented caseload. Moreover, the pragmatic approach we adopted to understand a novel disease resulted in the inclusion of patients with severe comorbidities and did not empower our study to assess the relationship between relevant interventions [e.g.: prone positioning or neuromuscular blockade]. and study outcomes. However, our multivariable regression analysis accounted for nuances related to the pandemic surge to produce a pragmatic assessment of which patients were more likely to die. Fourth, we limited the inclusion of severity scores in our study. For instance, we did not record SOFA score as most COVID-19 patients were admitted to ICU by uniquely scoring the respiratory component. In this setting, further organ compromise would gradually accrue accompanying the insidious progression of the disease. Instead, we recorded surrogate measures such as vasopressor use, P/ F ratio and requirement of RRT. Also, we did not include SAPS 3 score in our regression analysis as the use of a composite variable would limit the relative importance of each component and hinder the individualized assessment of relevant risk factors.

## Conclusions

We reported the clinical characteristics and outcomes of 215 patients receiving mechanical ventilation during the COVID-19 pandemic in Sao Paulo, Brazil. Also, we identified the main risk factors for mortality: age, creatinine at the intubation date, lower BMI, PF ratio, barotrauma and duration from symptom onset to intubation. These data are relevant to support clinical decision making and enable comparisons of outcomes in patients with COVID-19 receiving mechanical ventilation.
